# Enabling methods for community health mapping in developing countries

**DOI:** 10.1186/1476-072X-9-56

**Published:** 2010-10-29

**Authors:** Rashid Ansumana, Anthony P Malanoski, Alfred S Bockarie, Abu James Sundufu, David H Jimmy, Umaru Bangura, Kathryn H Jacobsen, Baochuan Lin, David A Stenger

**Affiliations:** 1 Njala University/Mercy Hospital Research Laboratory, Kulanda Town, Bo, Sierra Leone; 2 Center for Bio/Molecular Science and Engineering, Naval Research Laboratory, Washington, DC 20375 USA; 3 Department of Global and Community Health, George Mason University, Fairfax, Virginia 22030 USA

## Abstract

**Background:**

Spatial epidemiology is useful but difficult to apply in developing countries due to the low availability of digitized maps and address systems, accurate population distributions, and computational tools. A community-based mapping approach was used to demonstrate that participatory geographic information system (PGIS) techniques can provide information helpful for health and community development.

**Results:**

The PGIS process allowed for the rapid determination of sectional (neighborhood) boundaries within the city of Bo, Sierra Leone. When combined with data about hospital laboratory visits, a catchment area for one hospital in Bo could be established. A survey of households from within the catchment area determined that the average population per household (about 6 individuals) was similar to that found in the 2004 census. However, we also found that the average house was inhabited by more than one household, for an average of 17.5 inhabitants per residential building, which is critical information to know when estimating population size using remote imagery that can detect and enumerate buildings.

**Conclusions:**

The methods developed in this paper serve as a model for the involvement of communities in the generation of municipal maps and their application to community and health concerns.

## Background

Low-income countries would benefit significantly from the expanded use of Geographic Information Systems (GIS) in the analysis of disease distribution, since GIS could provide more accurate information about disease incidence and prevalence rates and would allow for better allocation of the limited resources available for public health [1-5]. However, these areas face significant barriers to the implementation of GIS for spatial epidemiology, due both to a lack of disease data (because of limitations in disease detection and reporting systems) and to the non-existence of detailed maps, especially in areas affected by conflict, population displacement, and rapid urbanization. Without accurate population, disease, and spatial data, it will not be possible to implement effective surveillance systems in these countries. Increasing the accuracy of these measures will require an improvement in data collection and management systems as well as a significant increase in the ability of scientists in low-income countries to apply epidemiological, laboratory, and GIS techniques to local health concerns [6-8].

Freely-available mapping and analysis tools and free or low-cost data and image sources (such as Google Earth) can be a greatly beneficial starting point for generating basic map features in areas where this information is not already available. However, effective disease surveillance requires that physical geographic information be supplemented by data about social and population factors that can be mapped at a fine scale. Combining information about distances from water, waste disposal areas, swampy areas, and other physical characteristics as well as information on population density, economic factors, and the location of health resources provides a more complete picture of health and disease by providing information about potential mitigating and debilitating factors. Even simple information about the location of patients' homes and the characteristics of those dwellings can provide helpful information about both physical and socioeconomic parameters that can be incorporated into a health GIS, information that is rarely available in low-income countries with limited access to technology. As the amount of remote imagery available for use in lower-income areas is expanding, so is the need for ground-truth validation of these high-resolution maps, which must be completed before advanced GIS analysis can be conducted [9-14].

Methods that engage and empower communities and allow local residents to incorporate their knowledge into the production of a GIS are a promising emerging approach for assembling ground truth data. Public Participation Geographic Information Systems (PPGIS) emerged in the 1980s as a formal method to involve the "public" in the creation and use of geographic information [15-17]. PPGIS and related approaches, such as participatory GIS (PGIS), community integrated GIS, and GIS for participation, have been adapted for use in a variety of settings with various levels of local participation, different types of communities, and a range of intended applications [17-20]. In each of these models, the goal is to integrate local knowledge with "expert" data and techniques.

In this study, we developed and used participatory methods to provide a low-cost solution to address mapping issues in Bo, Sierra Leone, and to begin populating a GIS with population statistics that can be used to address many community issues. Our specific aims were as follows: (1) to create a map of the sections (neighborhoods) of Bo by employing a participatory mapping method, which included interviews of knowledgeable long-term local residents and consultation with municipal authorities in the Bo City Council and local officials and (2) to estimate total population in a few sections by combining data from the map, the 2004 census, and population data obtained from household surveys. As part of the first aim and an example of how maps can be applied to health-related research, an analysis of visits to the Mercy Hospital Laboratory was performed using hospital records. Future studies will use the information from the map and surveys of households sampled from residential buildings identified on the map to assist with the initiation of an active infectious disease surveillance system and the analysis of the social and environmental factors contributing to the incidence and prevalence of diseases.

## Results

### Sectional mapping

Our first goal was to use a participatory process to create a map of the boundaries for each section within the city of Bo, which is the second largest city in Sierra Leone and the urban center of Bo District (the equivalent of a state or province). Although sections are formally-recognized and distinct areas of the city, no official map of the sections in Bo has been made since 1964, when the Directorate of Overseas Surveys (D.O.S.) produced a map of Bo with sectional information and street addresses for Sierra Leone's Ministry of Lands, Housing, Country Planning, Forestry and the Environment (MLCPE). This map is now obsolete due to the significant growth of the city over the past forty years and to a civil war from 1991 to 2002 that destroyed much of the country's infrastructure and capacities. Now that peace has been re-established, new maps are required to provide accurate current information. The Development Assistance Coordination Office (DACO) and the Sierra Leone Information System (SLIS) have developed a map of main roads and landmark buildings from 2002 Ikonos imagery, but this map does not show the location of the sections of Bo city.

**Figure 1:**
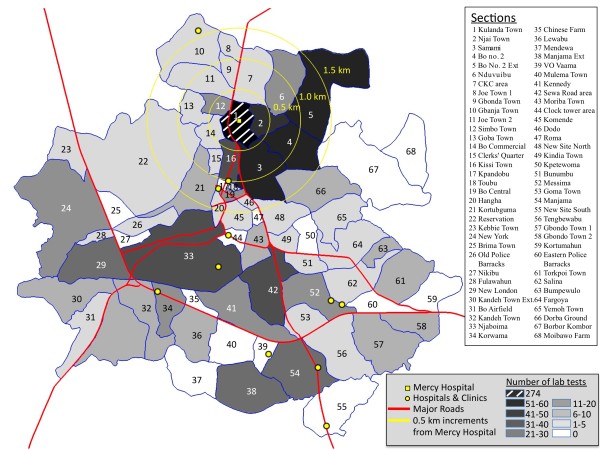
**Sectional map of Bo**. Bo, Sierra Leone, with sections and major roads indicated. The locatio of all hospitals and large clinics are marked with point. Concentric circles in the northern part of the city with 0.5 km, 1.0 km, and 1.5 km radii are marked to indicate distance from Mercy Hosptial (marked as small square) to various sections of the city. Fill color is indicative of the number of patients who had lab tests from each section.

The participatory process employed in this study resulted in the mapping of 68 sections within the 30.1 km^2 ^area encompassed by the city of Bo. Each section has a unique shape (Figure 1) and size (Table 1), with sectional areas ranging from 0.02 km^2 ^(Toubu) to 2.33 km^2 ^(Bo Government Reservation). The section map provides organizational information about the city of Bo, and additional information, such as the locations of hospitals and large clinics (Figure 1), can be displayed at the sectional level.

**Table 1:** Sections (neighborhoods) of Bo, sectional areas, and number of Mercy Hospital Laboratory patients

Location	**Area (km**^**2**^**)**	Visits	Location	**Area (km**^**2**^**)**	Visits
All	30.10	797	New Site North	0.32	4

Kulanda Town *	0.30	274	Fargoya	0.54	3

Samami^†^	0.59	55	CKC area^†^	0.46	3

Bo No. 2^†^	0.48	55	Hangha	0.09	3
		
Bo No. 2 Extension	1.08		Yemoh Town	0.40	3

Njai Town^†^	0.21	47	Kebbie Town	0.28	3

Kissi Town^†^	0.20	36	Salina	0.47	2

Sewa Raod area	0.64	33	Fulawahun	0.08	2

Njaboima	1.75	31	Kindia Town	0.15	2

New London	0.60	28	Clerks' Quarter^†^	0.08	2

Manjama	0.74	23	Komende	0.20	1
		
Manjama Extension	0.70		Dodo	0.05	1

Kennedy	0.64	19	Goma	0.27	1

New York	1.51	17	Bo Commercial^†^	0.18	1

Simbo Town^†^	0.14	16	Bo Airfield	0.43	1

Nduvuibu^†^	0.49	15	Tengbewabu	0.68	1

Messima	0.49	15	Old Police Barracks	0.23	1

Bo Central	0.07	13	Toubu	0.02	1

Korwama	0.30	11	New Site South	0.69	1

Kandeh Town	0.54	9	Bunumbu	0.18	1
		
Kandeh Town Extension	1.32		Brima Town	0.19	0

Kortubguma	0.52	9	VO Vaama	0.15	0

Dorba Ground	0.63	9	Kortumahun	0.35	0

Lewabu	0.48	8	Eastern Police Barracks	0.23	0

Moriba Town	0.25	6	Kpetewoma	0.20	0

Torkpoi Town	0.55	6	Nikibu	0.10	0

Bumpewulo	0.15	6	Kpandobu	0.03	0

Gbondo Town 1	0.72	5	Mulema Town	0.43	0
		
Gbondo Town 2	0.31		Roma	0.04	0

Reservation	2.33	5	Borbor Kombor	1.22	0

Goba Town^†^	0.21	5	Moibawo Farm	0.50	0

Gbanja Town	0.64		Chinese Farm	0.10	0
		
Gbonda Town^†^	0.12	4	Clock tower area	0.11	0
		
Joe Town 1	0.12		Mendewa	0.47	0
		
Joe Town 2^†^	0.42				

Note: The second column shows the areas of each section of Bo; the third column shows the number of people from each section who were tested at the Mercy Hospital Laboratory. (* denotes the section in which Mercy Hospital is located and ^† ^denotes sections with at least half their area within 1 km of Mercy Hospital. Some adjacent sections are grouped together for visit analysis.)

### Catchment area study

The sectional map will be useful for a variety of applications, including public planning and social and health research. A brief illustration of this applicability involves the use of the map to define a catchment area for one of the hospitals in Bo, Mercy Hospital, which is on the north side of the city (Figure 1). A summary of the residents of Bo who visited Mercy Hospital Laboratory for clinical testing and provided the name of their home section is shown in Table 1. (Patient information was extracted from the hospital's computerized database.) Figure 1 shows the home sections for those 797 patients (which represent only 52% of the 1810 total laboratory patients) using a darker grey to represent sections that are home to more Mercy Hospital patients. As might be expected, the majority of laboratory patients (n = 274) live in the section in which Mercy Hospital is located, Kulanda Town. The number of patients is smaller in other sections (n = 0 to 55), and the number decreases as the distance from the hospital becomes greater. The section map will make it easier for future patients to identify their home neighborhoods, which will increase the proportion of patients who can be included in the GIS.

Figure 1 also shows that 13 sections have half or more of their area located within 1 km of Mercy Hospital. From Table 1, it can be determined then that 509 (64%) of hospital patients with a known residential section live in one of these nearby sections, which represent only 12.5% (3.76 km^2^) of the total area of Bo. This demonstrates that the pool of people who normally visit Mercy hospital was strongly associated with distance from the hospital.

### Population estimation

Many public policy decisions and community health assessments are based on rates and other characteristics rather than just counts. The calculation of a rate requires an accurate numerator - a subset of the population that meets certain criteria - as well as an accurate denominator, such as the total population in a section of a city or the age- and/or sex-specific count of residents. The total number of residents can be projected from the number of houses in each section and the average household density of a section. The sectional map for Bo can serve as a baseline for estimating the current population of Bo, which is an essential step toward having an accurate "denominator" for the estimation of rates of disease and other characteristics of residents of Bo.

**Figure 2:**
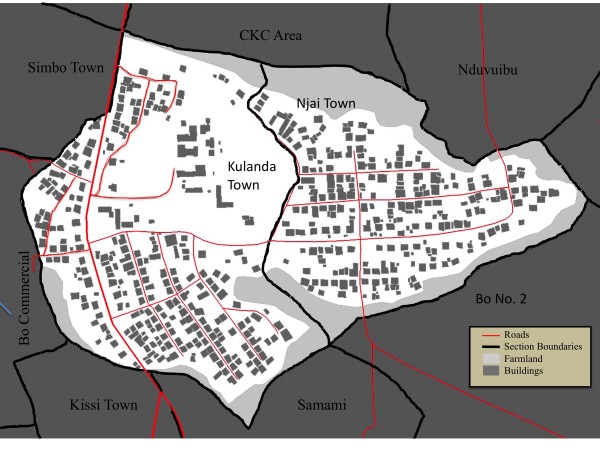
**Building structures in Kulanda Town and Njai Town**. Results of the Kulanda Town and Njai Town rooftop extraction (dark blue) with roads (red lines), section boundaries (black lines), and farmland (green areas). The dark grey boxes are the results of digitization of building rooftops from images.

A preliminary examination of population size was conducted in the two sections of Bo located nearest to Mercy Hospital, Kulanda Town and Njai Town. First, remote sensing was used to identify structures. Then, census data from 2004 was used to estimate the population size. Finally, a household survey was conducted to determine the total number of inhabited buildings and the total number of residents in each of the residential structures. These participatory methods could later be implemented in the other sections of Bo identified on the section map. Figure 2 shows the household map for Kulanda Town and Njai Town. There were 316 potential residential structures in Kulanda Town and 260 in Njai Town.

An estimate of population density per household was possible based on the estimated number of buildings shown in remote imagery and data from the 2004 national census data. In 2004, Bo District had a population of approximately 450,000, of which about 150,000 resided in the urban center of Bo [21,22]. The average number of residents per household ranged from 6.0 to 7.0 within the various census tracts within Bo [22]. If each building is home to one household and each has an intermediate household density of about 6.5 residents, then about 3700 individuals (2000 in Kulanda Town and 1700 in Njai Town) were estimated to reside in the two sections. However, this estimate was suspected to be inaccurate both because it is difficult to distinguish rooftops of residences from rooftops buildings used for other purposes and because of expected population growth between 2004 and 2010.

To obtain a better estimate of the current population density within houses in Bo, we conducted a census of Kulanda Town and Njai Town. As an initial step, the number of actual residential structures was determined to be 197 in Kulanda Town and 130 in Njai Town, which was less than the number of building rooftops identified from the remote imaging. The average household population size was 6.1 people. This was within the range of estimates from the 2004 national census. However, we found that many houses that would typically be classified as modest single-family residences were home to multiple semi-independent households (often composed of relatives who maintain separate sleeping and cooking areas). The 327 houses were home to 1027 households (637 in Kulanda Town and 390 in Njai Town) with a total of 6245 residents, for an average of 19.0 people per building (19.7 for Kulanda Town and 17.9 for Njai Town). The total number of residents in Kulanda Town is then not the 2000 estimated from the map and 2004 census data, but 3894, a striking difference.

Based on the new population estimate for the section, we estimate that 7.0% of Kulanda Town residents were tested at the Mercy Hospital Laboratory during the study period. Having a more accurate denominator for the rate significantly reduces the estimate of the proportion of residents of Kulanda Town who visited the laboratory for testing during the study period from 13.7% to 7.0%. For Njai Town, the total resident population was 2351, and only 2.4% of Njai Town residents were tested at Mercy Hospital Laboratory during the study period. This more accurate number is essential for health services planning. In this example, the lower proportion of the population being served by Mercy Hospital may provide evidence that the local population is underserved by health service providers. Additional survey work in other sections of Bo with varying numbers of residential structures and expected population densities will be required to better understand how to infer population size and density from remote sensing, and how to better define hospital catchment areas and utilization of health care resources.

## Conclusions

Participatory methods made possible the creation of detailed, accurate maps for Bo, Sierra Leone, and determined the population of two sections despite a very limited budget for the project. The importance of maps for public policy and other applications was highlighted by two simple demonstrations, the identification of a catchment area for one hospital and the use of the new sectional map to identify houses eligible to participate in a community census. This analysis showed that the majority of the hospital's patients reside within the area adjacent to the hospital and that the number of patients from a section decreases as the distance from the hospital increases. Some sections outside this area had non-negligible numbers of visits but determining the significance of this is not possible at this time since this study showed that population counts from the 2004 census were not accurate estimates of the current population. Those sections might have a high population density, which would mean that the rate of attendance at Mercy Hospital was not elevated compared to other sections equally distant from the hospital.

It is interesting to note that although we determined that the average population per household was similar to that found in the 2004 census, the average house was home to 3.1 households. Multiple households within one house could be caused by several factors alone or in combination: unavailability of housing in a rapidly urbanizing area, lack of affordability of housing due to demand that raises prices, or cultural factors that value living in close proximity to relatives and extending hospitality to family members in need. Further interaction with community informants could provide insight into the mechanisms at work in Bo.

The methods developed in this paper build on previous PGIS and PPGIS papers [15-20] and serve as a model for the involvement of communities in the generation of municipal maps and their application to social and health concerns. One of the key observations during field work is that community involvement is often the only way to acquire accurate information about boundaries in areas where residential areas often begin as informal settlements. This study demonstrated effective participatory methods to determine the population of a section and to identify geographic information about the section. Together these will improve the application of spatial epidemiology in low- and middle-income countries by allowing for the calculation of more accurate rates. This paper demonstrates that a participatory mapping approach can mitigate some of the challenges inherent to mapping in low-resource areas and can be a first step toward implementing GIS for public applications, such as disease surveillance, the determination of the preferred location of public services, and the management of future outbreaks.

## Methods

### Sectional mapping

As a first step toward generating a sectional map, we interviewed knowledgeable long-term local residents and, in consultation with municipal authorities in the Bo City Council and local officials of the MLCPE, sketched out the boundaries of each section. We then georeferenced these boundaries using GPSMAP 60Cx Hiking GPS receivers (Garmin International, Inc., Olathe, KS), to the extent that it was possible to do so given that some of the terrain involved physical obstacles such as swamps and that in some places trespassing laws did not allow us to access boundary areas. Main roads were also tracked. The raw GPS tracks were loaded into DNR Garmin software (MapSource-trip and waypoint manager), converted to KML format for intermediate processing. The KML file structure allows coordinates to be adjusted using a text editor and the results of the updates could be confirmed by viewing in Google Earth before final import into ArcMap.

We asked local elders who had resided within the section for at least 15 years to review the map and check our preliminary map boundaries for accuracy. We also received input from Bo city officials. Based on the feedback of both the elders and the city officials, we made further adjustments to the KML description of each section, including adjustments to coordinates so that all locations within the town fell into one defined section. A final review of the boundaries was then made by all participating parties. After our local experts had approved the boundaries, we used DNR Garmin software (longitude and latitude coordinates having UTM WGS84 Zone 29 reference data) to convert the section boundaries from the KML format into a shapefile. This shapefile was imported into ArcGIS. In ArcCatalog, a new polygon shapefile was created and dragged into the ArcMap content table. ArcMap was then used to create a digital polygon of the Bo municipal sections by tracing the boundary lines using the editor toolbar.

### Catchment area study

Patient records from Mercy Hospital and its Laboratory, a private medical facility located on the north side of Bo in the Kulanda Town section (Figure 1), were used for the assessment of catchment area. Mercy Hospital Laboratory maintains electronic records of all patients referred to it for testing. The records contain patient demographic data, such as age, sex, and home address (often recorded as only the section of residence when a street address is not available), as well as the results of the various tests that were conducted at the laboratory. These records were queried for all Mercy Hospital patients who were referred for testing to Mercy Hospital Laboratory between February 2009 and March 2010. When laboratory information about residential location was missing, it was filled in from the patient's outpatient records when available. To protect patient privacy, no personally identifiable information, such as patient names or addresses, was included in our search records, and our analysis in this paper is aggregated at the section level.

### Population estimation

The first task required to make an estimation of population in a section is to determine the number of houses in the section. Because software that automatically marks houses by identifying rooftops from remote imagery is, at present, quite expensive, we were unable to use this specialty software and had to digitize the location of homes manually. In ArcMap a new empty window was opened and given WGS 84 UTM Zone 29 reference data. Using the 'Add data' tool, a georeferenced satellite image was added to provide a background image for digitization. ArcCatalog was then opened and new shapefiles were created to represent buildings and streets/roads and given WGS84 UTM Zone 29 reference data. These shapefiles were then added to the ArcMap. The database table of the polygon shapefile representing buildings was opened and a new field added to contain the map section data within which a building was located. Using the ArcEditor tool, the shapefile of interest was selected and the Sketch Tool was used to create a footprint of a particular feature, using a line to represent streets and a polygon to represent buildings.

Once the buildings in each section were identified, an initial physical pass was made through the streets to note which buildings could clearly be identified as being used for purposes other than a residence, such as barns, sheds, and businesses. A notation was added to the map to indicate viable residences and these were assigned unique identifiers.

Every identified residence was visited and an adult resident asked to identify how many people lived in the household. (The household survey was approved by the research ethics committees of Njala University, Bo; George Mason University, Fairfax, Virginia, USA; and the U.S. Naval Research Laboratory, Washington, DC, USA.) The initial visits revealed that residents would usually report that multiple "households" were present within their homes. For all buildings visited, the query included the number of households living in the building and the number of individuals who were members of each household. Two households in Kulanda Town (99.7% participation rate) and five households in Njai Town (98.7% participation rate) declined to participate. The average household population was determined for each section from the households that participated. The estimated population was calculated as this average multiplied by the total number of households within the section. In a similar manner the total number of individuals per building was calculated only from buildings in which all households participated.

## Competing interests

The authors declare that they have no competing interests.

## Authors' contributions

RA was involved in study conception, survey collection, and drafting of the manuscript. APM was involved in the study conception, analysis of data, and drafting the manuscript. ASB was involved data collection and preparation of the sectional mapping and house mapping and contributed to the manuscript. AJS was involved data collection and preparation of the sectional mapping and house mapping. DHJ performed survey and patient information collection. UB performed survey and patient information collection and was involved in study conception. KHJ was involved in the design of the census and revising the manuscript critically for important intellectual content. BL was involved in revising the manuscript critically for important intellectual content. DAS was involved in study conception, manuscript revision and gave final approval of the version to be published. All authors have read and approve of the final manuscript.
